# MRI Volume Fusion Based on 3D Shearlet Decompositions

**DOI:** 10.1155/2014/469015

**Published:** 2014-04-10

**Authors:** Chang Duan, Shuai Wang, Xue Gang Wang, Qi Hong Huang

**Affiliations:** ^1^School of Electronic Engineering, University of Electronic Science Technology of China, Qingshuihe Campus, No. 2006, Xiyuan Avenue, West Hi-Tech Zone, Chengdu, Sichuan 611731, China; ^2^Research Institute of Electronic Science and Technology, University of Electronic Science Technology of China, Qingshuihe Campus, No. 2006, Xiyuan Avenue, West Hi-Tech Zone, Chengdu, Sichuan 611731, China; ^3^Electronic Engineering College, Chengdu University of Information Technology, No. 24, Section 1, Xuefu Road, Southwest Airport Economic Development Zone, Chengdu, Sichuan 610225, China

## Abstract

Nowadays many MRI scans can give 3D volume data with different contrasts, but the observers may want to view various contrasts in the same 3D volume. The conventional 2D medical fusion methods can only fuse the 3D volume data layer by layer, which may lead to the loss of interframe correlative information. In this paper, a novel 3D medical volume fusion method based on 3D band limited shearlet transform (3D BLST) is proposed. And this method is evaluated upon MRI T2* and quantitative susceptibility mapping data of 4 human brains. Both the perspective impression and the quality indices indicate that the proposed method has a better performance than conventional 2D wavelet, DT CWT, and 3D wavelet, DT CWT based fusion methods.

## 1. Introduction

Medical image fusion is a special case of image fusion and has been studied for decades. It is widely applied in medical diagnostics [1, 2]. It refers to extracting and merging the feasible information from different source images, which were captured by different kinds of sensors, such as CT, MRI, and PET, or different configurations of the same sensor, such as MRI T2* and quantitative susceptibility mapping (QSM). Some information is correlated, but most of the information is complementary, because special sensors or special configurations of the same sensor are sensible to special sources. For example, CT images provide the details of dense hard tissues, MRI images give information of soft tissues: T2* provides the contrast of the tissue relaxation time, and QSM can be provide susceptibility contrast information, which is produced by a range of endogenous magnetic biomarkers and contrast agents such as iron, calcium, and gadolinium (Gd). If different data can be properly fused, the fused data contain all the feasible information of the scanned object, which can reveal the details of structure more clearly than each single sensor. Previously, all source data need to be registered. Because 3D T2* magnitude image and QSM image are acquired from the real and imaginary part of the same scan, QSM images are exactly registered to T2* images.

Nowadays, many researches on medical fusion method only consider the 2D case. However, many medical sensors can provide 3D data volume, and the value of each point in the volume is correlated not only to the adjacent points in the same layer but also to the points in neighboring layers. Therefore, it is necessary to develop the volume fusion method instead of 2D image fusion method which can only fuse the data in single layer.

Fusion methods can be performed in spatial domain or certain transformed domain. In spatial domain, the intuitive fused image is selected as the weighted average image of source images. This kind of methods is relatively easy to implement, but the performances are low and some feasible information is reduced or even lost. The transformed domain of fusion methods is usually following the following steps: (1) registering source images, (2) performing the forward transform to sources images, (3) acquiring the fused coefficients from coefficients of source images under fusion rules, and (4) performing backward transform to fused coefficients to get the fused image. In this type of methods, the research works are usually focused on two points: the choice of the transform and the design of fusion rule. Many multiscale transforms are applied in fusion methods, such as DWT, DTCWT, curvelets [3], and shearlets [4].

Shearlets emerged in recent years among the most successful frameworks for the efficient representation of multidimensional data. Indeed, many other transforms were introduced to overcome the limitation of traditional multiscale transforms due to their poor ability of capturing edges and other anisotropic features. However, shearlet transform stands out since it has many advantages uniquely. It has a single or finite set of generating functions; it provides optimally sparse representations for multidimensional data; it allows a unified treatment of the continuum and digital realms. With these advantages, shearlet transform has been widely utilized in many image processing tasks such as denoising [5], edge detection [6], and enhancement [7]. And in many papers [4], as well as this paper, shearlet transform is indeed also very suited to image fusion. In this paper, the 3D band limited shearlet transform, which is the discrete implementation of shearlet transform, is selected for medical volume fusion.

Three fusion rules are utilized in this paper: maximum points' modulus (MPM), which considers only the value of single point; maximum regional energy (MRE), which considers the information for the local region [8] and treats each point of the region equally; and maximum sum of modified laplacian [9], which also considers the information in the region but treats the center point of the region and the points around it differently. Other more complicated fusion rules have also been proposed. The above three methods were selected as representatives. These three classic fusion rules are expanded into 3 dimensions. In order to evaluate the performance of proposed method, the quality indices also are extended into 3D version.

The rest of the paper is organized as follows. In Section 2, the basic theories about 3D shearlet transform and the discrete implementation, 3D BLST, are briefly introduced. In Section 3, fusion method based on 3D BLST with three fusion rules are proposed. Using the experiments of Section 4, the comparison of 2D and 3D methods and the performance of the proposed methods are illustrated and discussed. Finally, we draw conclusions in Section 5.

## 2. 3D Shearlet Transform

In this section, the basic theory of 3D shearlet transform and its discrete implementation, 3D band limited shearlet transform (3D BLST), are introduced.

### 2.1. Basic Theory of the 3D Shearlet Transform

**Figure 1:**
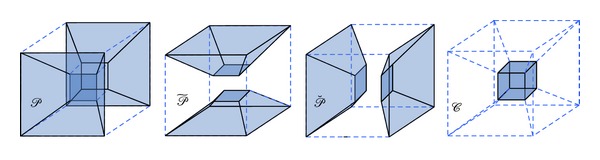
The partition of frequency domain for 3D pyramid-adapted shearlet transform.

As shown in Figure 1, the 3D frequency domain can be partitioned into three pairs of pyramids given by

(1) $$\begin{array}{c} 𝒫=\left\{\left(\xi_{1},\xi_{2},\xi_{3}\right)\in {\mathbb{R}}^{3}:\left|\xi_{1}\right|\geq 1,\left|\frac{\xi_{2}}{\xi_{1}}\right|\leq 1,\left|\frac{\xi_{3}}{\xi_{1}}\right|\leq 1\right\},\\ \tilde{𝒫}=\left\{\left(\xi_{1},\xi_{2},\xi_{3}\right)\in {\mathbb{R}}^{3}:\left|\xi_{2}\right|\geq 1,\left|\frac{\xi_{1}}{\xi_{2}}\right|\leq 1,\left|\frac{\xi_{3}}{\xi_{2}}\right|\leq 1\right\},\\ \overset{˘}{𝒫}=\left\{\left(\xi_{1},\xi_{2},\xi_{3}\right)\in {\mathbb{R}}^{3}:\left|\xi_{3}\right|\geq 1,\left|\frac{\xi_{1}}{\xi_{3}}\right|\leq 1,\left|\frac{\xi_{2}}{\xi_{3}}\right|\leq 1\right\} \end{array}$$

and the center cube

(2) $$\begin{array}{c} 𝒞=\left\{\left(\xi_{1},\xi_{2},\xi_{3}\right)\in {\mathbb{R}}^{3}:||\left(\xi_{1},\xi_{2},\xi_{3}\right)||<1\right\}. \end{array}$$

The partitioning of frequency space into pyramids allows restricting the range of the shear parameters. Without such partitioning, one must allow arbitrarily large shear parameters, which leads to a treatment biased toward one axis. The defined partition, however, enables restriction of the shear parameters to [⌈−2^*j*/2^⌉, ⌈2^*j*/2^⌉]. This approach is the key to provide an almost uniform treatment of different directions in a sense of a good approximation to rotation.

Pyramid-adapted shearlets are scaled according to the paraboloidal scaling matrices as *A*_2^*j*^_, ${\tilde{A}}_{2^{j}}$ and ${\overset{˘}{A}}_{2^{j}}$, *j* ∈ *ℤ* defined by $A_{2^{j}}=\left(\begin{array}{ccc} 2^{j} & 0 & 0\\ 0 & 2^{j/2} & 0\\ 0 & 0 & 2^{j} \end{array}\right)$, ${\tilde{A}}_{2^{j}}=\left(\begin{array}{ccc} 2^{j/2} & 0 & 0\\ 0 & 2^{j} & 0\\ 0 & 0 & 2^{j} \end{array}\right)$, and ${\overset{˘}{A}}_{2^{j}}=\left(\begin{array}{ccc} 2^{j} & 0 & 0\\ 0 & 2^{j} & 0\\ 0 & 0 & 2^{j/2} \end{array}\right)$. Directionality is encoded by the shear matrices *S*_*k*_, ${\tilde{S}}_{k}$, and ${\overset{˘}{S}}_{k}$, *k* = (*k*_1_, *k*_2_) ∈ *ℤ* ^2^, given by $S_{k}=\left(\begin{array}{ccc} 1 & k_{1} & k_{2}\\ 0 & 1 & 0\\ 0 & 0 & 1 \end{array}\right)$, ${\tilde{S}}_{k}=\left(\begin{array}{ccc} 1 & 0 & 0\\ k_{1} & 1 & k_{2}\\ 0 & 0 & 1 \end{array}\right)$, and ${\overset{˘}{S}}_{k}=\left(\begin{array}{ccc} 1 & 0 & 0\\ 0 & 1 & 0\\ k_{1} & k_{2} & 1 \end{array}\right)$, respectively. The translation lattices will be defined through the following matrices: *M*_*c*_ = diag⁡(*c*_1_, *c*_2_, *c*_2_), ${\tilde{M}}_{c}=\mathrm{diag}(c_{2},c_{1},c_{2})$, and ${\overset{˘}{M}}_{c}=\mathrm{diag}(c_{2},c_{2},c_{1})$, where *c*_1_ > 0 and *c*_2_ > 0. Then the definition of 3D pyramid-adapted discrete shearlet can be given as follows.

**Definition 1**

For *c* = (*c*_1_, *c*_2_)∈(ℝ_+_)^2^, the pyramid-adapted discrete shearlet system $\text{SH}(\phi ,\psi ,\tilde{\psi },\overset{˘}{\psi };c)$ generated by $\phi ,\psi ,\tilde{\psi },\overset{˘}{\psi }\in L^{2}({\mathbb{R}}^{3})$ is defined by

(3) SH(ϕ,ψ,ψ~,ψ˘;c)=Φ(ϕ;c1)∪Ψ(ψ;c)∪Ψ~(ψ~;c)∪Ψ˘(ψ˘;c),

where Φ(*ϕ*; *c*_1_) = {*ϕ*_*m*_ = *ϕ*(·−*m*) : *m* ∈ *c*_1_ *ℤ* ^3^},

(4) Ψ(ψ;c)={ψj,k,m=2jψ(SkA2j·−m)≥0,|k|≤⌈2j/2⌉,m∈Mcℤ3},Ψ~(ψ~;c)={ψ~j,k,m=2jψ~(S~kA~2j·−m)≥0,|k|≤⌈2j/2⌉,m∈M~cℤ3},Ψ˘(ψ˘;c)={ψ˘j,k,m=2jψ˘(S˘kA˘2j·−m)≥0,|k|≤⌈2j/2⌉,m∈M˘cℤ3}, j∈ℕ0,  k∈ℤ2.

### 2.2. 3D Band Limited Shearlet Transform

3D band limited shearlet transform (3D BLST) is one discrete implementation of 3d pyramid-adapted shearlet transform. Let the shearlet generator *ψ* ∈ *L* ^2^(ℝ^3^) be defined by $\hat{\psi }(\xi )={\hat{\psi }}_{1}(\xi_{1}){\hat{\psi }}_{2}(\xi_{2}/\xi_{1}){\hat{\psi }}_{2}(\xi_{3}/\xi_{1})$, where *ψ*_1_ and *ψ*_2_ satisfy the following assumptions:

- ${\hat{\psi }}_{1}\in C^{\infty }(\mathbb{R})$, $\mathrm{supp}{\hat{\psi }}_{1}\subset [-4,-1/2]\cup [1/2,4]$, and $\sum_{j\geq 0}^{}{\left|{\hat{\psi }}_{1}(2^{-j}\xi )\right|}^{2}=1$ for |*ξ*| ≥ 1, *ξ* ∈ ℝ.
- ${\hat{\psi }}_{2}\in C^{\infty }(\mathbb{R})$, $\mathrm{supp}{\hat{\psi }}_{1}\subset [-1,1]$, and $\sum_{l=-1}^{1}{\left|{\hat{\psi }}_{2}(\xi +l)\right|}^{2}=1$ for |*ξ*| ≤ 1, *ξ* ∈ ℝ.

Thus, in frequency domain, the band limited shearlet function *ψ* ∈ *L* ^2^(ℝ^3^) is almost a tensor product of one wavelet with two “bump” functions and, thereby, a canonical generalization of the classical band limited 2D shearlets. This implies the support in frequency domain by a needle-like shape with the wavelet acting in radial direction and ensures high directional selectivity. The derivation from a tensor product in fact ensures a favorable behavior with respect to the shearing operator and thus a tiling of frequency domain which leads to a tight frame for *L* ^2^(ℝ^3^).

**Theorem 2**

Let *ψ* be a band limited shearlet defined as before; the family of functions *P*_*𝒫*_Ψ(*ψ*) forms a tight frame for ${\dot{L}}^{2}(𝒫):=\left\{f\in L^{2}({\mathbb{R}}^{3}):\mathrm{supp}\hat{f}\subset 𝒫\right\}$, where *P*_*𝒫*_ denotes the orthogonal projection onto ${\dot{L}}^{2}(𝒫)$ and Ψ(*ψ*) = {*ψ*_*j*,*k*,*m*_ : *j* ≥ 0, |*k*| ≤ ⌈2^*j*/2^⌉, *m* ∈ (1/8)*ℤ* ^3^}.

By this theorem and a change of variables, the shearlet tight frames for ${\dot{L}}^{2}(𝒫)$, ${\dot{L}}^{2}(\tilde{𝒫})$, and ${\dot{L}}^{2}(\overset{˘}{𝒫})$ can be constructed, respectively. Furthermore, wavelet theory provides choice of *ϕ* ∈ *L* ^2^(ℝ^3^) such that Φ(*ϕ*; 1/8) forms a tight frame for ${\dot{L}}^{2}(𝒞)$. Since ${\mathbb{R}}^{3}=𝒞\cup 𝒫\cup \tilde{𝒫}\cup \overset{˘}{𝒫}$ as a disjoint union, any function can be expressed by *f* ∈ *L* ^2^(ℝ^3^) as $f=P_{𝒞}f+P_{𝒫}f+P_{\tilde{𝒫}}f+P_{\overset{˘}{𝒫}}f$, where *P*_*C*_ denotes the orthogonal projection onto the closed subspace ${\dot{L}}^{2}(C)$ for some measurable set *C* ⊂ ℝ^3^. More details of 3D shearlet theory and the implementation of band limited shearlet transform as well as other implementations can be found in [10–14].

## 3. Proposed Fusion Method

The proposed fusion method in this paper belongs to the voxel-level fusion, with average rule for low frequency coefficients and three different fusion rules for high frequency coefficients.

*(a) Max Modulus of Points' Modulus (MPM). *One has

(5) $$\begin{array}{c} C_{f}=\{\begin{array}{cc} C_{a}, & \left|C_{a}\right|\geq \left|C_{b}\right|\\ C_{b}, & \left|C_{a}\right|<\left|C_{b}\right|. \end{array} \end{array}$$

The fused high coefficients are the coefficients that have the larger modulus as represented in (5), where *C*_*t*_, *t* ∈ {*a*, *b*, *f*} means the high frequency coefficients; *a*, *b* label two sources, respectively; *f* refers to the fused result. This fusion rule considers only the point information.

*(b) Max Region Energy (MRE) [8]. *One has

(6) $$\begin{array}{c} C_{f}=\{\begin{array}{cc} C_{a}, & \left|E_{a}\right|\geq \left|E_{b}\right|\\ C_{b}, & \left|E_{a}\right|<\left|E_{b}\right|, \end{array} \end{array}$$

where $E_{t}=\left(1/N_{\Omega }\right)\sum_{p\in \Omega }^{}\sqrt{{\left(C_{t}(p)-{\overset{-}{C}}_{t}\right)}^{2}}$, *t* ∈ {*a*, *b*}, *Ω* is a local region, ${\overset{-}{C}}_{t}$ is the mean of all *C*_*t*_ in *Ω*, and *N*_*Ω*_ is the number of coefficients in *Ω*. The fused high coefficients are the coefficients that have the larger local energy. This kind of method considers not only the information of the current position but also that information around it.

*(c) Max Region Sum of Modified Laplacian (MSML).* The fused high frequency coefficients are acquired according to (7). 3D version of modified Laplacian index is calculated through (9), and the sum of them is calculated as (8); *Ω* is a local region. In this paper, the variation step equals 1:

(7) $$\begin{array}{c} C_{f}=\{\begin{array}{cc} C_{a}, & \text{SM}L_{a}\geq \text{SM}L_{b}\\ C_{b}, & \text{SM}L_{a}<\text{SM}L_{b}, \end{array} \end{array}$$

(8) $$\begin{array}{c} \text{SML}\left(i,j,k\right)=\underset{p,q,t\in \Omega }{\sum }{\left[\text{ML}\left(i+p,j+q,k+t\right)\right]}^{2}, \end{array}$$

(9) ML(i,j,k) =|2I(i,j,k)−I(i−step,j,k)−I(i+step,j,k)|  +|2I(i,j,k)−I(i,j−step,k)−I(i,j+step,k)|  +|2I(i,j,k)−I(i,j,k−step)−I(i,j,k+step)|.

**Figure 2:**
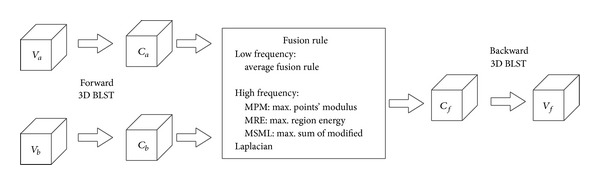
Steps of proposed medical volume fusion method.

The steps of proposed fusion method are given in Figure 2. Firstly, 3D BLST are performed to both source volumes; the low frequency is the average of both source coefficients, the low frequency coefficients are the average of both low frequency coefficients of source images, the high frequency coefficients are acquired by equations (5)–(9). Finally, the backward 3D BLST is performed to fused coefficients, and the output is the fused volume as represented by *V*_*f*_.

## 4. Experiment

In this section, the performances of proposed methods are evaluated on 4 human brain subjects. The human study was approved by our Institutional Review Board. MR examinations were performed with a 3.0T MR system (Signa HDxt, GE, USA), using an 8-channel head coil. A 3D T2* weighted multiecho gradient echo sequence was used with the following parameters: FA = 20°; TR = 57 ms; number of TEs = 8; first TE = 5.7 ms; uniform TE spacing (ΔTE) = 6.7 ms; BW = ±41.67 kHz; field of view (FOV) = 24 cm; a range of resolutions were tested: 0.57 × 0.75 × 2 mm^3^. The 3D T2* magnitude and QSM images reconstructed by NMEDI [15] are interpolated to 128 × 128 × 128 for fusion. Because in QSM processing the magnetic field outside the brain parenchyma was corrupted by noise, QSM region was cropped by a mask, which was obtained using a brain extraction tool (BET) [16]. In following comparisons, the fusion regions are performed in the mask.

### 4.1. 2D versus 3D: The Consistency along the* z*-Axis

The volume data has 3 dimensions, that is, *x*-, *y*-, and *z*-axis. In the first experiment, the 2D methods are performed along *x*- and *y*-axis frame by frame. And the 3D methods directly fuse the whole volume data. One major difference between these two type methods is the treatment of data along *z*-axis. The consistency along *z*-axis is compared by the visual effect of inter frame difference (IFD) images and the measurement of IFD_MI [17, 18]. When calculating IFD_MI, only the voxel which located in both masks of frames is calculated, because the data out of the mask is invalid and the mask is different in IFD images. Suppose the IFD_MI is acquired by MI_*i*_(*D*_*a*_(:, :, *i*), *D*_*b*_(:, :, *i*), *D*_*f*_(:, :, *i*)), where *D*_*a*_, *D*_*b*_, *D*_*f*_ refer to the IFD images from the source *V*_*a*_, *V*_*b*_ and fused volume *V*_*f*_, *D*_*d*_(:, :, *i*) = *V*_*d*_(:, :, *i* + 1) − *V*_*d*_(:, :, *i*), *d* ∈ {*a*, *b*, *f*}. The IFD_MI of this paper is calculated by (10), where *N* refers to the number of frames along *z*-axis and • means pointwise multiplication:

(10) IDF_MI=1N−1∑i=1N−1(MIi(Da(:,:,i),Db(:,:,i),Df(:,:,i))•(Mask(:,:,i+1)∪Mask(:,:,i))).

**Figure 3:**
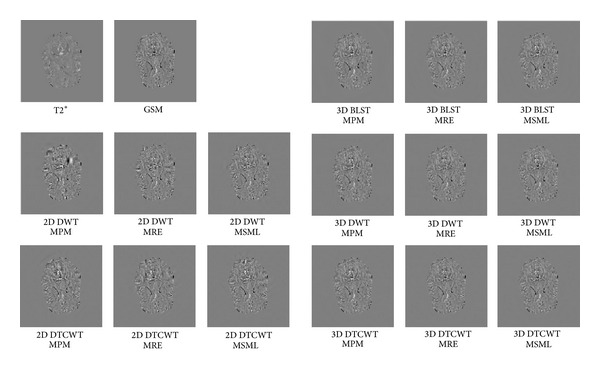
Interframe differences for 2D and 3D fusion methods.

From the visual impression of the IFD images (Figure 3), it can be noticed that the fused images by 2D methods have several obvious distortions which make the fused images similar to neither of the source images. While, in results by 3D methods, the IDF images are more consistent with the IDF of source data, which means the IDF images are highly correlated to the IDF of source images (Figure 3). The IDF images for fused volumes are very similar to the IDF of QSM data. And the difference among the 3D methods can hardly be noticed. This conclusion can still be drawn from the quality index of IFD_MI, as given in Tables 1, 2, 3, and 4. The 3D BLST with MPM fusion has the highest value of IFD_MI, and all the values for 3D methods are higher than that of the 2D methods with the same fusion rule.

### 4.2. Performance of Proposed Methods

In the second experiment, the visual effect and the quality index are compared among the fusion methods based on 2D, 3D DWT, 2D, 3D DTCWT, and 3D BLST. Two widely used performance indices are selected as the subjective measurements of the fused results: mutual information (MI) and *Q* ^AB∣*F*^. However, in document [19], the index of *Q* ^AB∣*F*^ is in only suited for the case of 2D images; it is necessary to be expanded into 3D in our experiment, where the 2D “Sobel” operator was substituted by 3D “Sobel” operator and the number of angels increases to 3. In most of the previous image fusion research, the quality index is calculated through the whole voxels (or pixels) of the source images (or volumes). However, in this experiment, only the points which located in the area of mask are taken into consideration, because those points outside the mask carry no useful information about the brains and are set to arbitrary value manually; consequently they are ignored in evaluation step.

**Figure 4:**
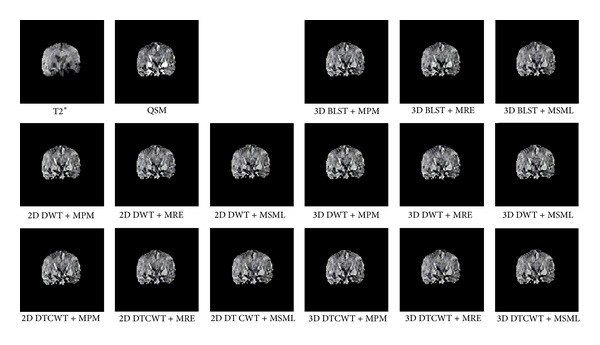
Coronal source and result images.

**Figure 5:**
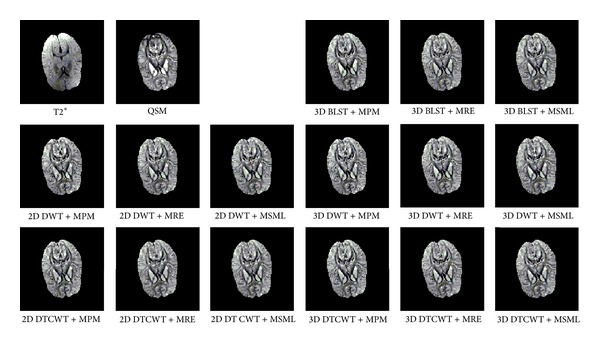
Axial source and result images.

**Figure 6:**
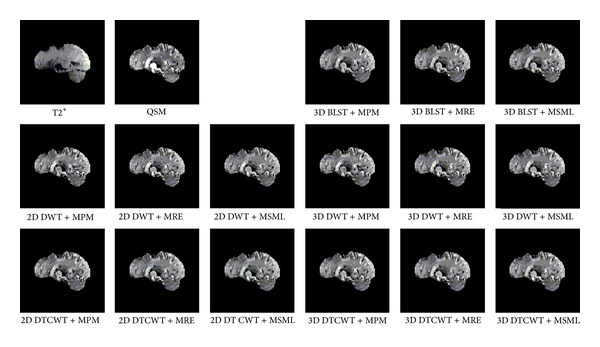
Sagittal source and result images.

One layer of each coronal, axial, and sagittal plane is selected as the representation; the source and result images are shown in Figures 4, 5, and 6. From the perspective impression, it is hard to tell which fusion method is better, because the resulting images are more similar to each other. The distinctions among them can only be noticed after carefully observation. This phenomenon suggests that the proposed method and all conventional methods can fulfill the fusion task effectively. The performance of different fusion methods can be compared through the quality indices which are listed in Tables 5, 6, 7, and 8. From the tables, it can be noticed that the quality indices of proposed method are larger than the methods based on DWT or DT CWT. And in the case of 3D BLST, among different fusion rules, the rule of MRE has the largest indices.

The method of this paper belongs to the voxel-level fusion method, which considers only the distribution of the shearlet coefficients. In the future, the inner structure feature of the organ will be taken into account to see whether it can further improve the quality of the fused medical volume.

## 5. Conclusion

In this paper, the 3D medical volume fusion method based on 3D band limited shearlet transform is proposed. From the principles of methods and the experiments the following conclusion can be drawn: (1) the 3D transform based methods have better consistency along *z*-axis (the third dimension) than conventional 2D transform based methods in medical volume fusion. (2) From both perspective impression and the quality indices, the proposed 3D BLST medical fusion method is better than 3D DWT or 3D DTCWT. (3) Among the fusion rules using 3D BLST, the MRE fusion rule has better performance than the other two fusion rules of MPM and MSML.

## Figures and Tables

**Table 1:** IFD_MI for the first group data.

IFD_MI	2D DWT	2D DTCWT	3D DWT	3D DTCWT	3D BLST
MPM	1.8443	1.7659	1.8905	2.2147	**2.5317**
MRE	1.7349	1.7650	1.8989	2.1558	2.3629
MSML	1.7274	1.7503	2.0965	2.3432	2.3766

**Table 2:** IFD_MI for the second group data.

IFD_MI	2D DWT	2D DTCWT	3D DWT	3D DTCWT	3D BLST
MPM	2.4516	2.4852	2.5762	2.8131	**3.0744**
MRE	2.4126	2.4130	2.4601	2.6169	2.8617
MSML	2.4105	2.4055	2.6021	2.7699	2.8555

**Table 3:** IFD_MI for the third group data.

IFD_MI	2D DWT	2D DTCWT	3D DWT	3D DTCWT	3D BLST
MPM	1.5034	1.5546	1.6884	2.0240	**2.3175**
MRE	1.4809	1.4978	1.6354	1.9907	2.1224
MSML	1.4705	1.4830	1.7859	2.0380	2.1244

**Table 4:** IFD_MI for the fourth group data.

IFD_MI	2D DWT	2D DTCWT	3D DWT	3D DTCWT	3D BLST
MPM	1.8390	1.9122	2.0275	2.3374	**2.6479**
MRE	1.8620	1.8957	2.0668	2.3143	2.5054
MSML	1.8573	1.8855	2.2373	2.4615	2.4778

**Table 5:** Performance of the first group.

First group		2D DWT	2D DTCWT	3D DWT	3D DTCWT	3D BLST
MPM	MI	1.1652	1.2168	1.1615	1.2404	1.2683
	*Q* ^AB∣*F*^	0.1824	0.1985	0.1820	0.2109	0.2288

MRE	MI	1.1596	1.2323	1.1471	1.2561	**1.2974**
	*Q* ^AB∣*F*^	0.1987	0.2185	0.1975	0.2351	**0.2611**

MSML	MI	1.1566	1.2339	1.2043	1.2617	1.2799
	*Q* ^AB∣*F*^	0.1977	0.2158	0.2257	0.2402	0.2497

**Table 6:** Performance of the second group.

Second group		2D DWT	2D DTCWT	3D DWT	3D DTCWT	3D BLST
MPM	MI	1.1897	1.2351	1.1840	1.2770	1.2960
	*Q* ^AB∣*F*^	0.2122	0.2326	0.2154	0.2555	0.2732

MRE	MI	1.2505	1.2921	1.2491	1.3271	**1.3340**
	*Q* ^AB∣*F*^	0.2461	0.2596	0.2498	0.2885	**0.3051**

MSML	MI	1.2545	1.2969	1.3056	1.3157	1.3232
	*Q* ^AB∣*F*^	0.2434	0.2578	0.2720	0.2927	0.2971

**Table 7:** Performance of the third group.

Third group		2D DWT	2D DTCWT	3D DWT	3D DTCWT	3D BLST
MPM	MI	0.9239	0.9661	0.9207	0.9915	1.0246
	*Q* ^AB∣*F*^	0.1763	0.1923	0.1785	0.2116	0.2283

MRE	MI	0.9514	1.0068	0.9592	1.0390	**1.0674**
	*Q* ^AB∣*F*^	0.1906	0.2084	0.2001	0.2333	**0.2546**

MSML	MI	0.9511	1.0087	1.0116	1.0605	1.0650
	*Q* ^AB∣*F*^	0.1872	0.2052	0.2241	0.2424	0.2457

**Table 8:** Performance of the fourth group.

Fourth group		2D DWT	2D DTCWT	3D DWT	3D DTCWT	3D BLST
MPM	MI	1.0968	1.1612	1.0960	1.1899	1.2168
	*Q* ^AB∣*F*^	0.1953	0.2170	0.1972	0.2363	0.2540

MRE	MI	1.1177	1.1879	1.1422	1.2283	**1.2512**
	*Q* ^AB∣*F*^	0.2157	0.2402	0.2354	0.2780	**0.2936**

MSML	MI	1.1225	1.1912	1.1959	1.2367	1.2401
	*Q* ^AB∣*F*^	0.2152	0.2367	0.2623	0.2860	0.2883
